# A Simple and Cost-Effective TLC-Densitometric Method for the Quantitative Determination of Acetylsalicylic Acid and Ascorbic Acid in Combined Effervescent Tablets

**DOI:** 10.3390/molecules23123115

**Published:** 2018-11-28

**Authors:** Alina Pyka-Pająk, Małgorzata Dołowy, Wioletta Parys, Katarzyna Bober, Grażyna Janikowska

**Affiliations:** Department of Analytical Chemistry, School of Pharmacy with Division of Laboratory Medicine in Sosnowiec, Medical University of Silesia in Katowice, Jagiellońska 4, 41-200 Sosnowiec, Poland; mdolowy@sum.edu.pl (M.D.); bober@sum.edu.pl (K.B.); gjanikowska@sum.edu.pl (G.J.)

**Keywords:** acetylsalicylic acid, ascorbic acid, TLC-densitometry, validation

## Abstract

A new, simple, and cost-effective TLC-densitometric method has been established for the simultaneous quantitative determination of acetylsalicylic acid and ascorbic acid in combined effervescent tablets. Separation was performed on aluminum silica gel 60F_254_ plates using chloroform-ethanol-glacial acid at a volume ratio of 5:4:0.03 as the mobile phase. UV densitometry was performed in absorbance mode at 200 nm and 268 nm for acetylsalicylic acid and ascorbic acid, respectively. The presented method was validated as per ICH guidelines by specificity, linearity, accuracy, precision, limit of detection, limit of quantification, and robustness. Method validations indicate a good sensitivity with a low value of LOD and LOQ of both examined active substances. The linearity range was found to be 1.50–9.00 μg/spot and 1.50–13.50 μg/spot for acetylsalicylic and ascorbic acid, respectively. A coefficient of variation that was less than 3% confirms the satisfactory accuracy and precision of the proposed method. The results of the assay of combined tablet formulation equal 97.1% and 101.6% in relation to the label claim that acetylsalicylic acid and ascorbic acid fulfill pharmacopoeial requirements. The developed TLC-densitometric method can be suitable for the routine simultaneous analysis of acetylsalicylic acid and ascorbic acid in combined pharmaceutical formulations. The proposed TLC-densitometry may be an alternative method to the modern high-performance liquid chromatography in the quality control of above-mentioned substances, and it can be applied when HPLC or GC is not affordable in the laboratory.

## 1. Introduction

Acetylsalicylic acid (ASA) belongs to a group of commonly used non-steroidal anti-inflammatory drugs. Besides the anti-inflammatory activity, it is used in the prevention of cardiovascular disease in low doses. Often the combined preparations of ASA and ascorbic acid (AA) are applied to relieve pain and fever. In view of the fact that leading pharmaceutical components used in these problems, there is a big need to develop a rapid and effective analytical method to simultaneous determine them in combined pharmaceutical formulations [1]. 

So far, various analytical techniques have been developed for the determination of ASA in pharmaceutical tablets such as UV-spectrophotometry [2], spectrofluorometry [3], HPLC (high-performance liquid chromatography) [4], and TLC (thin-layer chromatography) [3,5,6,7,8]. The method recommended by Polish and United States Pharmacopoeias for ASA is HPLC with UV detection [9,10]. 

A number of described methods are suitable for the determination of ASA in biological samples, e.g., in blood [3,11,12,13,14,15], skin [12], and urine [14,15]. The main advantage of these methods is the speed and satisfactory results of validation parameters.

For example, Patel and coworkers [6] used TLC to simultaneously determine the ASA in clopidogrel hydrogen sulfate in tablets. The analysis was carried out on plates precoated with silica gel 60F_254_ using a mobile phase composed of ethyl acetate-methanol-toluene-glacial acetic acid (5:1:4:0.1, *v/v*/*v/v*). Validation of the method showed its high accuracy (recovery was 99.93%), precision (RSD = 0.279%), and sensitivity (LOD = 82.86 ng/mL, LOQ = 276.21 ng/mL). Franeta et al. [7] analyzed the tablets, containing ASA, paracetamol, caffeine, and phenobarbital. The tests were carried out on silica 60F_254_ plates by use of a mixture of dichloromethane-ethyl acetate-cyclohexane-isopropanol-0.1M HCl-fumaric acid (9:8:3:1.5:0.2:0.2, *v/v/v/v/v/v*) as the mobile phase and were estimated by densitometric scanning. The results showed the high accuracy of the method (recovery value: 98.5%) and good precision (RSD = 0.58–0.95%). In addition to this, the described technique allowed the separation of salicylic acid (SA).

Krzek and coworkers [16] have analyzed the tablets containing ASA and methocarbamol using the TLC method. The separation was performed on silica gel plates by means of the mobile phase: methyl acetate, methanol, and 25% ammonia in volume composition 17:2:1. The results obtained were accurate and reproducible, however, they did not allow for the separation of SA.

The second studied active compound, namely AA, has been previously determined in pharmaceutical preparations by the following methods: iodometric [17], spectrophotometric [18,19], flow analysis technique [20,21], ion chromatography [22], LC-MS/MS [23], and TLC [24,25]. The method recommended by Polish and United States Pharmacopoeias for AA is titration [9,10]. 

A literature review also reveals that the methods which determine the ascorbic acid in food, e.g., in milk [26] as well as in biological samples, i.e., in human plasma [23,27]. 

Jarzębiński and Ługowska [24] have examined AA in combined pharmaceutical preparations containing various substances including salicylamide, rutoside, and caffeine using the TLC method. The analysis was carried out on silica gel plates by means of a mobile phase chloroform-ethanol-water (1:2:0.5, *v/v/v*). The results were comparable with those obtained by the iodometric method. 

The TLC combined with densitometry was also useful for the determination of AA and dipyrone in a pure form as well as a pharmaceutical dosage forms such as tablets and ampoules [25]. The separation was carried out on silica gel 60F_254_ plates using a mobile phase composed of water and methanol (95:5, *v/v*). The high accuracy (recovery was in the range of 98.3–98.7%), good precision (RSD = 1.8–2.3%), and high sensitivity (1.25 μg/mL) characterized this technique [25]. 

The next paper confirms the utility of modern chromatographic techniques like ultra-performance liquid chromatography (UPLC) with tandem mass spectrometry (MS/MS) [1], hydrophilic interaction liquid chromatography (HILIC) [28], and HPLC with a photodiode detector [29] for the determination of both AA and ASA in the pharmaceutical dosage form. 

The UPLC with MS/MS detection was successfully applied for the simultaneous determination of AA and ASA in effervescent tablets. Chromatographic analysis was performed by means of a C18 column and a mixture of 0.1% formic acid in water and acetonitrile in a ratio of 75:25 (*v/v*). The developed method was characterized by a high accuracy (recovery > 99%), good precision (RSD = 1.3–2.6%), and high sensitivity (LOD < 0.09 μg/g). The time of the analysis was less than 2 min [1].

In the case of the HILIC analysis of AA, ASA, and SA, a proper Zorbax RX-SIL column was used and mobile phase consisted of acetonitrile and 22 mM ammonium acetate (82:18, *v/v*). This method was validated in terms of linearity, accuracy (RSD < 5%), and precision. However, the limit of detection for AA and ASA has not been determined [28].

The separation of AA and ASA in effervescent tablets was also determined by HPLC with a photodiode detector and Betasil C18 column. A mixture of water and acetonitrile with 0.1% formic acid (75:25, *v/v*) was applied as the mobile phase. The validated parameters were very satisfactory. The recovery was greater than 98% (precision (RSD < 2.1%) and sensitivity expressed as LOD and LOQ were 0.00051–0.0014 mg/mL) [29]. 

It is important to highlight that the TLC and HPLC techniques can analyze the same compounds. Planar chromatography has many analytical advantages. The analysis of the sample conducted by the TLC technique is equal to 20% of the costs which would be incurred by analyzing this sample by the HPLC or GC techniques. In the case of TLC, there is no need for tedious and time-consuming sample preparation and the removal of impurities or possible interferences which is the case most of the time HPLC and GC. The large volume of the sample investigated can be spotted on a chromatographic plate. Thus, the samples do not have to be concentrated in many cases. Several samples may be simultaneously spotted and separated on one plate. For these reasons, the TLC has a relatively rapid chromatographic technique. Moreover, various detection methods can be used in TLC for the same substances analyzed and it raises the analytical values of the results obtained in many cases.

The current literature review of the pharmaceutical use of TLC in drug analyses indicates that the modern thin layer chromatography with quantitative densitometric TLC procedures are still frequently used in quality and quantity control as a rapid and low-cost method with simple sample preparation. However, according to our knowledge, until now, there has been no official TLC-densitometric method for the simultaneous determination of ASA and AA using a simple mobile phase.

In our papers, we have previously investigated the optimum conditions for the separation of caffeine, ethoxybenzamide, and acetylsalicylic acid from its related substance, and salicylic acid by normal phase thin layer chromatography (NP-TLC) with densitometry [5,8]. It was ascertained that the best conditions to separate acetylsalicylic acid from salicylic acid enables the mobile phase: n-hexane-diethyl ether-acetic acid (80%) in a volume ratio of 7:2:1 [5]. The second mixture of *n*-hexane-acetone-glacial acetic acid in a volume composition of 7 mL:2 mL:1 drop was suitable for the separation of caffeine, ethoxybenzamide, and acetylsalicylic acid from its related substance [8]. These conditions were used with component tablets such as *Aspirin Protect, Polopiryna S, Cardiopirin* and *Acard* as well as for the assay of acetylsalicylic acid, caffeine, and ethoxybenzamide in combined tablets like *Etopiryna* and *Coffepirine* (trade names of the drugs in Poland). The described TLC methods were validated by specificity, range, linearity, accuracy, precision, limit of detection, limit of quantification, and robustness. 

The present work is a continuation of our previous papers and aimed at the development of proper chromatographic conditions for the separation of acetylsalicylic acid, ascorbic acid, and salicylic acid, as well establishing a quantitative assay for the determination of acetylsalicylic acid and ascorbic acid in marketed effervescent tablets by the TLC-densitometric method with regard to obligatory validation [30,31].

## 2. Results and Discussion

### 2.1. Validation of TLC Method

**Figure 1:**
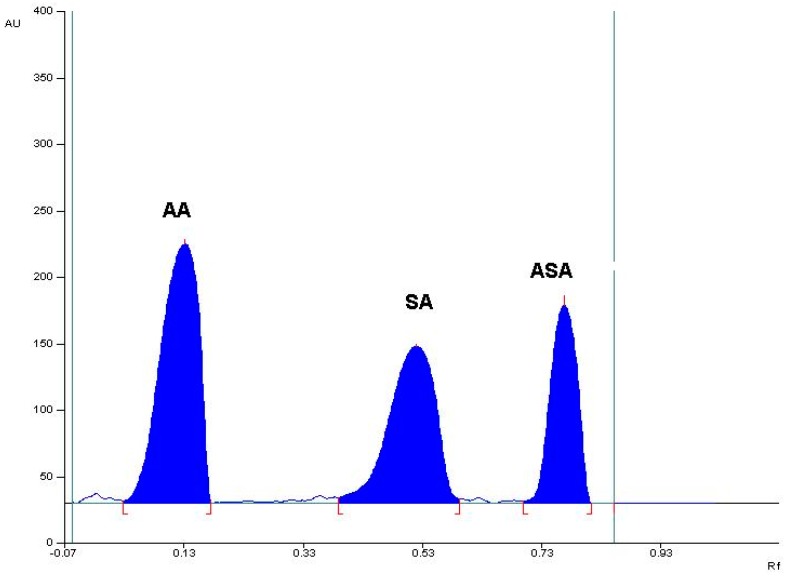
The densitogram obtained from ascorbic acid (AA), acetylsalicylic acid standards (ASA) spiked with a related substance to ASA, namely salicylic acid (SA) (mobile phase B4: chloroform: ethanol 96%: glacial acetic acid (5:4:0.03, *v/v/v*)).

**Figure 2:**
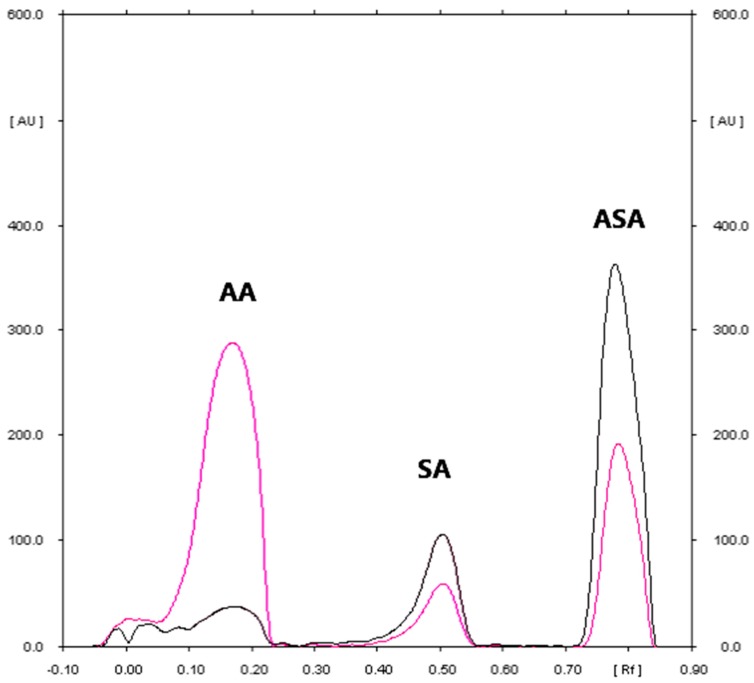
The densitogram of ascorbic acid (AA), salicylic acid (SA), and acetylsalicylic acid standard (ASA) coming from commercial effervescent tablets sample (Densitometric scanning were performed at absorption maximum equal to 200 nm and 268 nm for ASA (black line) and AA (pink line)).

**Figure 3:**
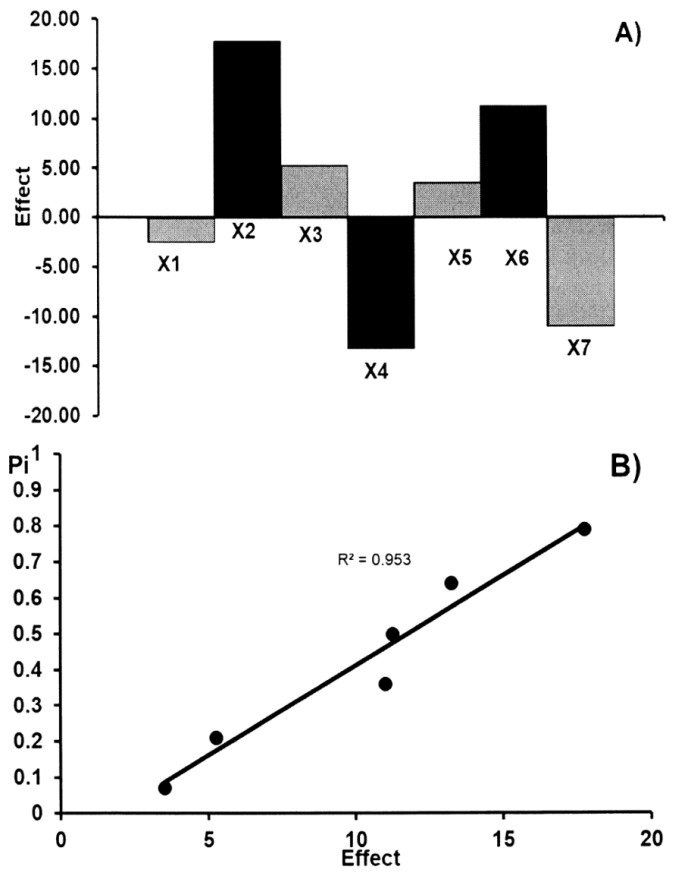
The robustness test: the effects of factors (**A**) and half-normal probability plot of effects (**B**) for the determination of acetylsalicylic acid in commercial effervescent tablets.

**Figure 4:**
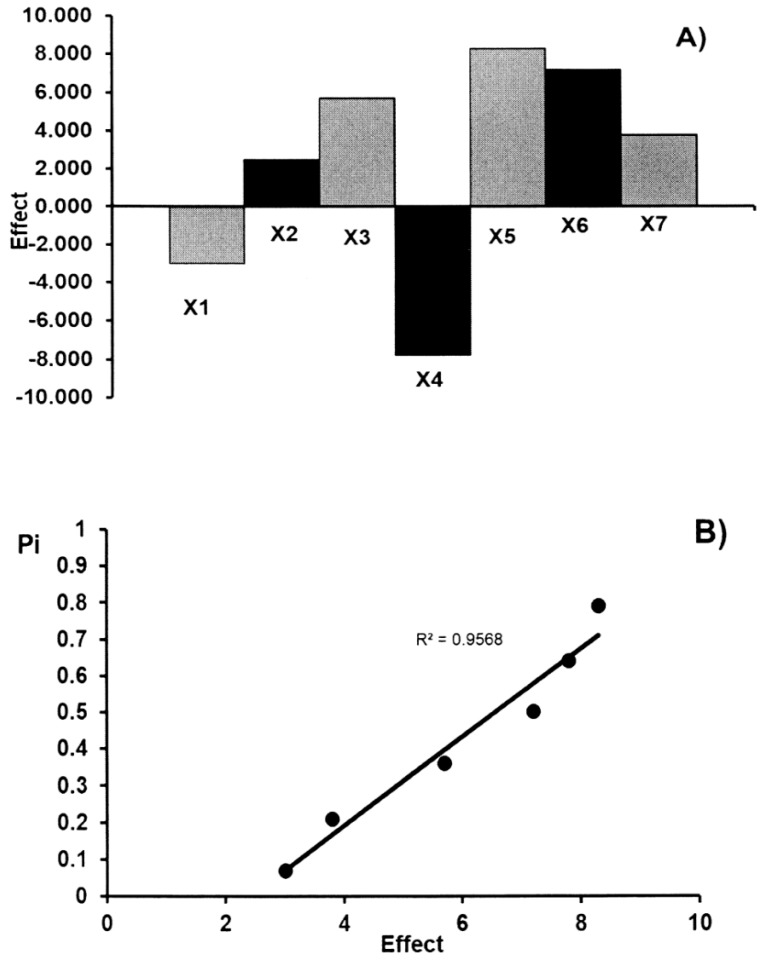
The robustness test: the effects of factors (**A**) and half-normal probability plot of effects (**B**) for the determination of ascorbic acid in commercial effervescent tablets.

The summarized results of the validation method are presented in Figure 1, Figure 2, Figure 3 and Figure 4 as well as in Table 1, Table 2, Table 3, Table 4 and Table 5, and consequently in the next subsubsections.

#### 2.1.1. Specificity

The separations of AA, SA, and ASA were achieved by choosing the optimal conditions for the TLC analysis. To select the one that will enable the best separation of the above-mentioned substances, the following (12 mobile phases) were tested:

- mobile phase A: glacial acetic acid:acetone:methanol:benzene (0.5:0.5:2:7, *v/v*/*v/v*) [32]
- mobile phase B: chloroform:ethanol 96%:water (3:6:1.5, *v/v/v*) [24]
- mobile phase B1: chloroform:ethanol 96% (3:6, *v/v*)
- mobile phase B2: chloroform:ethanol (5:5, *v/v*)
- mobile phase B3: chloroform:ethanol 96%:glacial acetic acid (5:5:0.03, *v/v/v*)
- mobile phase B4: chloroform:ethanol 96%:glacial acetic acid (5:4:0.03, *v/v/v*)
- mobile phase C: methylene chloride:ethyl acetate:ethanol 96% (5:5:1, *v/v/v*) [33]
- mobile phase D: *n*-hexane:diethyl ether:80% acetic acid (7:2:1, *v/v/v*) [5]
- mobile phase E: *n*-hexane:acetone:glacial acetic acid (5:5:0.09, *v/v/v*)
- mobile phase F: *n*-hexane:acetone:glacial acetic acid (5:5:0.5, *v/v/v*)
- mobile phase G: acetone:*n*-hexane:glacial acetic acid (5:5:0.06, *v/v/v*)
- mobile phase G1: *n*-hexane-acetone-glacial acetic acid in the following volume composition: 7 mL:2 mL:1 drop [8].

The preliminary study with the use of the mentioned mobile phases indicated that the mobile phases A, B, and F did not ensure a satisfactory separation of SA from ASA. In the case of the mobile phases examined—C, D, E, G, and G1—AA remained on the start line. The mobile phases B1 and B2 caused a weak separation of SA from ASA. Mobile phases B3 and B4 allowed a good separation of the tested reference substances, but the mobile phase B4 enabled us to obtain the best relation between the R_F_ values for AA, SA, and ASA, enabling the successful separation, as well as identification, of all the compounds tested. Therefore, a mixture of chloroform, ethanol (96%), and glacial acetic acid in a volume ratio of 5:4:0.03 was chosen as the mobile phase. An exemplary TLC densitogram of separation of the examined mixture (ASA, AA, and SA) is shown in Figure 1. In the case of this mobile phase, the following R_F_ values were noted: R_F(AA)_ = 0.18 ± 0.05, R_F(SA)_ = 0.56 ± 0.03, R_F(ASA)_= 0.80 ± 0.03. The calculated separation factor (R_S_) for R_S(AA/SA)_ = 2.93, and R_S(SA/ASA)_ = 2.15 also confirms the suitability of the selected mobile phase for the separation of the studied compounds. The spectro-densitometric analysis showed that the AA basic band occurs at 268 nm, SA at 299 nm, and ASA at 200 nm.

The densitogram of AA and ASA (Figure 2) indicates that the TLC technique combined with densitometry used to quantify AA and ASA in *Polopiryna C* (produced by Polpharma) is highly selective. The mean values of the R_F_ of AA and ASA were 0.18 and 0.80, respectively and they were consistent with the R_F_ value which was achieved for both active substances coming from the tablet extracts. Similarly, the spectro-densitograms of AA and ASA standards are similar to those derived from the studied tablet samples. What is important is that the proposed method also allows for the detection of SA in pharmaceutical preparations. Just like other papers [5,7,34], during the initial step of the conducted studies, the presence of a small amount of SA in the examined sample can be observed. Over time, the amount of SA can increase slightly at the expense of ASA. It is probably caused by the partial hydrolysis of the examined SA. For this fact, the determination of ASA in simple as well as combined pharmaceutical preparations should be carried out immediately after its extraction from the pharmaceutical sample. 

#### 2.1.2. Accuracy

The accuracy of the method was evaluated by the measurement of recovery (Table 1 and Table 2). When known amounts of acetylsalicylic acid and ascorbic acid were added to powdered tablets containing a proper amount of acetylsalicylic acid and ascorbic acid, the recoveries for ASA and AA from commercial effervescent tablets were placed in the range of 99.0–99.8% and 99.3–100.3% (Table 1 and Table 2). The low coefficient of variation values (CV < 3%) are indicative of the accuracy of the method.

#### 2.1.3. Calibration and Range

The statistical data shown in Table 1 and Table 2 indicate that a good linear relationship exists between the measured peak area (AU) and the concentrations of acetylsalicylic acid and ascorbic acid (μg/spot). The plots were linear in the following ranges: 1.50 to 13.50 μg/spot, and 1.50 to 9.00 μg/spot for ascorbic acid and acetylsalicylic acid, respectively. The graphs of the residuals against the concentrations of acetylsalicylic acid and ascorbic acid were also plotted. It can be observed that the residuals were distributed above and below the zero residuals line, thus, confirming the linearity of the proposed TLC method. 

#### 2.1.4. Precision

The precision (repeatability and intermediate) of the proposed method was studied at three different concentrations of ASA and AA from the tablet extractions. The results from these experiments expressed as the coefficients of variation (CV, %) of the respective response factors (a relationship between the peak area and concentrations of acetylsalicylic acid and ascorbic acid, respectively) are presented in Table 1 and Table 2. Because the CV values for repeatability and intermediate precision were <3%, the method was precise.

#### 2.1.5. Limit of Detection (LOD) and Limit of quantification (LOQ) Based on the Calibration Curves

The limits of detection and quantification were 0.20 μg/spot and 0.61 μg/spot, and 0.25 μg/spot and 0.75 μg/spot for acetylsalicylic acid and ascorbic acid, respectively. The low LOD and LOQ values confirm the sensitivity of the proposed method. 

#### 2.1.6. Robustness

The main effects of the seven factors were tested on two levels in eight experiments (Table 3). Table 4 shows the results obtained for the acetylsalicylic acid and ascorbic acid contents (*y_i_*) in commercial effervescent tablets. These results show that no factor has a significant effect on the results. These data were also evaluated by the half-normal probability plotting of the rank probabilities (*p_i_*) as a function of the absolute values of the main effects. The effects of factors and the half-normal probability plot of effects for the determination of acetylsalicylic acid and ascorbic acid in commercial effervescent tablets are presented in Figure 3 and Figure 4, respectively. The points of all factors are placed near the straight line, which indicates that their effect is negligible (R^2^ > 0.953). Therefore, the presented TLC-densitometric method can be regarded as robust. The standard deviation of acetylsalicylic acid and ascorbic acid contents (*y_i_*) in commercial effervescent tablets with the seven parameters which have been changed in the conducted experiment in order to check the robustness of the applied method was 3.01% and 4.18% for ASA and AA, respectively. The value of CV (<5%) indicates the reliability of the proposed TLC-densitometric method, but the content of the glacial acetic acid in the mobile phase should be constant.

#### 2.1.7. Analysis of Acetylsalicylic Acid and Ascorbic Acid in Commercial Effervescent Tablets

In each case, the R_F_ values of acetylsalicylic acid and ascorbic acid standards, and those coming from commercial effervescent tablets were equal to 0.18 ± 0.02, and 0.80 ± 0.02, respectively. The identities of acetylsalicylic acid and ascorbic acid standards from the samples (commercial effervescent tablets) were also confirmed by analysis of their spectra. A very good correspondence between both spectro-densitograms was stated. In all cases, the absorption maxima (λ_max_) were equal to 200 nm, and 268 nm for acetylsalicylic acid and ascorbic acid, respectively. The purities of the peaks of acetylsalicylic acid and ascorbic acid standards the and samples were also assessed by comparing the spectra obtained from acetylsalicylic acid and ascorbic acid standards at the peak start (S), peak apex (M), and peak end (E) of the spot. It was found that r(S,M) > 0.999 and r(M,E) > 0.999 for all analyses performed by the proposed TLC-densitometric technique. Statistical data showing the results of the quantitative determination of acetylsalicylic acid and ascorbic acid obtained on the basis of ten repeated different analyses of pharmaceutical preparations are presented in Table 5. It was stated that acetylsalicylic acid and ascorbic acid amounts in commercial effervescent tablets which were determined by the TLC-densitometric method are equal to 97.1%, and 101.6% in relation to the label claim. It is in agreement with the percentage content required by the Polish and American Pharmacopoeias for acetylsalicylic acid and ascorbic acid as active ingredients [9,10].

### 2.2. Comparison with the Indirect Iodometric Method

To verify the results of ascorbic acid in commercial effervescent tablets obtained by the proposed TLC-densitometric method, a comparison of the TLC results with previous reports concerning indirect iodometric method was made [17]. When compared with the indirect iodometric method for ascorbic acid tablets, similar results were obtained for ten different repeated analyses (Table 5). The average assay of ascorbic acid was found to be 203.2 ± 1.4 mg/tablet and 201.3 ± 1.8 mg/tablet for the TLC-densitometric and indirect iodometric methods, respectively. The coefficients of variance were smaller than 3% in both cases. High reproducibility and insignificant differences between the two compared methods were obtained at the 95% probability level from the *t*-test and F-test of significance at 1.89 < 2.101 and 1.64 < 3.18 for commercial effervescent tablets. These results confirmed that the TLC-densitometric method is accurate and can be used as a substitute method for the quantitative analysis of ascorbic acid. 

## 3. Materials and Methods

### 3.1. Apparatus

Densitometer: Camag (Muttenz, Switzerland) TLC Scanner 3 with the winCATS 1.4.2 software (Camag, Muttenz, Switzerland). IKA Ultra-Turrax® Tube Drive Workstation (IKA Poland Sp. z o.o., Warsaw, Poland) with BMT-20-S Tube for grinding with balls of stainless steel. NP-TLC plates: 10 × 20 cm glass plates precoated with 0.20-mm layers of silica gel 60F_254_ (E. Merck, Darmstadt, Germany, # 1.05554); 10 × 20 cm aluminum plates precoated with 0.20 mm layers of silica gel 60F_254_ (E. Merck, Germany, # 1.05570). The 5 μL Camag micropipettes (Muttenz, Switzerland) were used to apply the solutions to the plates.

Chromatographic chamber: a classical chamber for 20 × 20 cm plates (#0.222.5259, Camag, Muttenz, Switzerland), and twin-trough chamber for 20 × 10 cm plates (#0.222.5254, Camag, Muttenz, Switzerland).

### 3.2. Chemicals

Acetylsalicylic acid (>99%, Sigma-Aldrich, Saint Louis, MO, USA), acetylsalicylic acid (USP grade, Sigma-Aldrich, USA), salicylic acid (>99%, Sigma-Aldrich, USA), ascorbic acid (>99%, Sigma-Aldrich, USA), were used as standards. All chemicals and reagents for TLC and iodometric methods were analytical grade and were purchased from POCh (Gliwice, Poland). 

### 3.3. Pharmaceutical Preparation

Pharmaceutical preparation, namely, *Polopiryna C* (Polpharma, Starogard Gdański, Poland) containing acetylsalicylic acid (500 mg) and ascorbic acid (200 mg) in one commercial effervescent tablets used in this study. 

### 3.4. Preparation Sample of Tablets

Ten tablets were ground for 30 min with a speed equal to 4000 rpm using an IKA Ultra-Turrax® Tube Drive Workstation with a BMT-20-S tube for grinding with balls of stainless steel. After this time, the obtained powders of commercial effervescent tablets (equivalent to 125 mg acetylsalicylic acid (ASA), and 50 mg ascorbic acid (AA) by weighing the powder with an accuracy of 0.1 mg) were extracted using 12 mL of ethanol absolute (99.8%) for 30 min with a speed equal to 4000 rpm using an IKA Ultra-Turrax® Tube Drive Workstation. After extraction, the solutions were filtered through a medium-density filter (Filtrak 389, Niederschlag, Germany) to volumetric flasks (25 mL) and replenished with the use of ethanol absolute to demanded volume. By the use of the obtained extracts, the following working solutions at the concentration of active substance equal to 8.00 mg, 4.80 mg, and 1.60 mg in 5 mL for acetylsalicylic acid, and 12.80 mg, 7.36 mg, and 2.26 mg in 5 mL for ascorbic acid were prepared. A total of 5 µL of each solution was used for the TLC-densitometric analysis and quantitative determination of acetylsalicylic acid and ascorbic acid in the examined commercial effervescent tablets. 

### 3.5. Preparation of Standard Solutions

Standard solutions of acetylsalicylic acid and ascorbic acid, as well as salicylic acid, were prepared by dissolving the solutes in absolute ethanol (99.8%).

### 3.6. Thin Layer Chromatography

Normal phase thin layer chromatography (NP-TLC) was done on TLC silica gel 60F_254_ plates (E. Merck, Darmstadt, Germany, # 1.05554). Additionally, the TLC silica gel 60F_254_ plates (E. Merck, Germany, # 1.05570) were used for the robustness test. The plates were prewashed with methanol and dried for 24 h at room temperature. Before use, the plates used in NP-TLC were activated at 120 °C for 10 min. 

The solutions of pharmaceutical samples and standards of active substances (5 μL) were spotted manually on the chromatographic plates. The mixture of the chloroform–ethanol (96%)-glacial acetic acid in a volume composition of 5:4:0.03 (*v/v/v*) was used as the mobile phase. A total of 50 mL of the mobile was used in all cases. After saturation of the chamber (20 cm × 20 cm) with the mobile phase vapor for 20 min, the plates were developed vertically at room temperature (20 °C) to a distance of 7.5 cm and then dried for 20 h at room temperature (20 °C) in a fume cupboard. Additionally, a twin-trough chamber of 20 × 10 cm (#0.222.5254, Camag, Muttenz, Switzerland) was used for the robustness test.

### 3.7. Densitometric and Spectro-Densitometric Study

Both densitometric and spectro-densitometric measurements were conducted by a TLC Scanner 3 (Camag, Switzerland) operated in the absorbance mode and controlled by the winCATS 1.4.2 software. The radiation source was a deuterium lamp emitting a continuous UV spectrum between 190 and 450 nm. Densitometric scanning was then performed at multi-wavelength in the range of 200 to 400 nm, at wavelength intervals of 50 nm for each step. Finally, the densitometric scanning was performed at an absorption maximum equal to 200 nm and 268 nm for acetylsalicylic acid and ascorbic acid, respectively. The slit dimensions were 12.00 × 0.40 mm (macro). The optimal optical system was light; the scanning speed was 20 mm/s; the data resolution was 100 μm/step; the measurement type was remission; and the measurement mode was absorption.

The chromatographic bands obtained on the densitograms were investigated by the spectro-densitometric analysis under the following conditions: the slit dimensions were 12.00 × 0.40 mm (macro); the optimal system was the resolution; the scanning speed was 20 nm/s; the data resolution was 1 nm/step; the initial wavelength was 200 nm, and the final wavelength was 400 nm; the measurement type was remission; and the measurement mode was absorption.

### 3.8. Validation of the NP-TLC Method

The proposed NP-TLC-densitometric method was validated by specificity, linearity, accuracy, precision, limit of detection, limit of quantification, and robustness according to the ICH guidelines [30] and according to the guidelines described by Ferenczi-Fodor et al. [31].

#### 3.8.1. Specificity

The specificity of the method was verified by the chromatography of working standards (acetylsalicylic acid, ascorbic acid) and salicylic acid as a related substance to acetylsalicylic acid, and a sample solution of acetylsalicylic acid and ascorbic acid extracted from tablets.

#### 3.8.2. Linearity and Range

The linearity of the TLC method was evaluated by the analysis of standard solutions of acetylsalicylic acid and ascorbic acid at concentrations of 2.700, 2.400, 2.000, 1.800, 1.500, 1.200, 0.900, 0.600, 0.300, 0.180, 0.090, and 0.044 mg/mL. The solutions (5 μL) were applied to the same plate. The plates were developed using above-mentioned mobile phases (described in the thin layer chromatography section) and scanned. The experiments were performed in six different analyses.

#### 3.8.3. Accuracy

This parameter was evaluated by the measurement of recovery. A proper amount of acetylsalicylic acid and ascorbic acid standards in the low, medium, and high regions of the calibration plots were added to powdered tablets of a known active substance content. Next, the tablets were extracted and analyzed under the optimized conditions. The experiments were performed in six different analyses.

#### 3.8.4. Precision

Intra-day precision of the method was verified by the analysis of three replicates of the three sample solutions (ethanol extracts of acetylsalicylic acid and ascorbic acid) at different concentrations under the same chromatographic conditions over a short interval of time (the same day). Inter-day precision was obtained for three sample solutions at different concentrations by an analyst who performed the analysis over a period of two weeks. To determine the precision of the procedure, the concentrations were prepared independently and the experiments were performed in three different analyses. The precision of the developed method was evaluated as the relative standard deviation (coefficient of variation, CV (%)).

#### 3.8.5. Limit of Detection and Limit of Quantification Based on the Calibration Curves

Specific calibration curves were studied using samples containing acetylsalicylic acid and ascorbic acid in the range of the limit of detection, namely 0.180, 0.088, and 0.044 μg/spot for acetylsalicylic acid, and 0.300, 0.180, and 0.090 μg/spot for ascorbic acid. The experiments were performed in six different analyses. The limit of detection (LOD) was calculated as follows:

(1) $$LOD=\frac{3.3\;\sigma }{S}$$

The limit of quantification (LOQ) was calculated as follows:

(2) $$LOQ=\frac{10\;\sigma }{S}$$

where, *σ* was the standard deviation of the response and *S* was the slope of the calibration curve.

#### 3.8.6. Robustness

The robustness test was prepared according to the guidelines described in the papers by Nagy-Turák and Ferenczi-Fodor et al. [31,35,36]. The robustness of the method was checked by spotting the sample solutions onto the plate and developing the plate after altering the conditions (Table 3). The conditions changed were the chamber type, the sorbent type, the temperature of plate activation, extraction time, the volume of chloroform in the mobile phase, the volume of ethanol in the mobile phase, and saturation time of the chamber. The method conditions and the selected factors with the values of their (+) and (−) levels are summarized in Table 3. A high level is represented by “+” and a low level by “−”. The main effects of the seven factors were tested on two levels in eight experiments [31,35]. The levels of the factors investigated and the experimental design matrix (2^3^) are shown in Table 3 and Table 4, respectively. The ways of calculation of the effects (*E*) characterizing the particular individual factors and rank probabilities [37] were presented earlier [5,8,30,35,36,38].

### 3.9. Comparing with the Indirect Iodometric Method

Developing a new analytical method for the determination of ascorbic acid in effervescent tablets required the comparison of the obtained results with other methods, e.g., the indirect iodometric technique [17]. For the indirect iodometric determination of vitamin C (ascorbic acid) in a pharmaceutical preparation, a copper (II) chloride was used. This method is based on the titration of the test sample with a CuCl_2_ solution in the presence of iodide excess. The test samples were prepared by dissolving 5 tablets in distilled water. The whole solution was diluted to 100 mL with distilled water. From the prepared solutions, 4 mL of it were taken accurately from 20 mL flasks and then 4 mL of potassium iodide solution, 1 mL of acetic acid, and 0.1 mL of starch as indicators were added and distilled water was added to the final volume. Samples were titrated with a CuCl_2_ solution to observe a permanent blue color. As a control, a direct titration with a solution of I_2_ was performed. Both methods were characterized by similar accuracy. An advantage of the described method is the stability of the copper (II) chloride solution as the titrant [17].

The comparison of the proposed TLC-densitometric method with the indirect iodometric method to determine ascorbic acid in pharmaceutical preparation was studied by the use of ten independently repeated different analyses. Student’s *t*-test was used to check the significance of the differences between the two analytical methods.

Because the series has the same number, the value of the parameter t was calculated using the following formula:

(3) $$t=\frac{\left|\bar{x_{1}}-\bar{x_{2}}\right|}{\sqrt{s_{1}^{2}+s_{2}^{2}}}\cdot \sqrt{n}$$

where, $\bar{x_{1}}$, $\bar{x_{2}}$ are the average values; s_1_, s_2_ are the calculated standard deviations for the results obtained using the two methods compared; *n* is the number of results.

The F-Snedecor value was also calculated using the following formula:

(4) $$F=\frac{s_{1}^{2}}{s_{2}^{2}}$$

where, s_1_, s_2_ were the calculated standard deviations for the results obtained using the two methods compared, on the assumption that s_1_ > s_2_. 

### 3.10. Statistical Analysis

Statistical evaluation of the obtained results was prepared by Statistica v 12.0 PL (StatSoft, Kraków, Poland).

## 4. Conclusions

The proposed new, simple, and cost-effective NP-TLC-densitometric method is suitable for rapid and cost-effective qualitative, as well as quantitative, analysis of acetylsalicylic acid and ascorbic acid in combined effervescent tablets. The described method fulfills all ICH guideline requirements in terms of validation. It is specific, sensitive, accurate, precise, and robust. The linearity range was found to be 1.50–9.00 μg/spot and 1.50–13.50 μg/spot for acetylsalicylic and ascorbic acid. 

The results of the assay of the combined tablet formulations equal 97.1% and 101.6% for acetylsalicylic acid and ascorbic acid (%) in relation to the label claim are in good agreement with the pharmacopoeial requirements. 

It could be said that the developed TLC-densitometric method can be used for the routine simultaneous analysis of acetylsalicylic acid and ascorbic acid in combined pharmaceutical formulations. 

Elaborated in this work, the TLC-densitometric method for the determination of acetylsalicylic acid and ascorbic acid in combined effervescent tablets is more accurate and precise than HILIC [28] and HPLC with the photodiode detector [29] methods, and just as accurate and precise as the UPLC with MS/MS detection method [1]. The results in our work statistically confirmed that the TLC-densitometric method is accurate and can be used as a substitute method for the assay of acetylsalicylic acid and ascorbic acid in combined pharmaceutical formulations, for example, in the case when HPLC or GC is not affordable in the laboratory.

## Figures and Tables

**Table 1:** The method validation data for the quantitative determination of ascorbic acid by NP-TLC (normal phase thin layer chromatography) with a densitometry ^a^.

Method Characteristic	Results	
Specificity	Specific	
Range (μg/spot)	1.50–13.50	
Linearity	A = 370.9 (±310.4) + 1944.8 (±36.8)·x *n* = 9; r = 0.999; s = 427.3; F = 2797.0; *p* < 0.0001	
Limit of detection (LOD) (μg/spot)	0.25	
Limit of quantification (LOQ) (μg/spot)	0.75	
**For Tablets**		
**Accuracy and Precision**		
Accuracy (*n* = 6)		
for 50% standard added (*n* = 6)	R = 100.3%; CV = 2.59%	
for 100% standard added (*n* = 6)	R = 99.3%; CV = 2.62%	
for 150% standard added (*n* = 6)	R = 100.0%; CV = 1.81%	
	Quantity of Precision (CV, (%)) *n* = 3	Ascorbic Acid
Interday	12.80 μg/spot	1.78
	7.36 μg/spot	1.38
	2.56 μg/spot	2.61
Intraday	12.80 μg/spot	1.77
	7.36 μg/spot	1.68
	2.56 μg/spot	2.94
Robustness	Robust	

^a^ A = peak area (AU), x = amount (μg/spot) of drug analyzed, r = correlation coefficient, R = recovery (%), CV = coefficient of variation (%).

**Table 2:** The method validation data for the quantitative determination of acetylsalicylic acid by NP-TLC with densitometry ^a^.

Method Characteristic	Results	
Specificity	Specific	
Range (μg/spot)	1.50–9.00	
Linearity	A = 2547.7 (±292.4) + 1532.7 (±50.0)·x *n* = 6; r = 0.9979; s = 314.0; F = 937.9; *p* < 0.0001	
Limit of detection (LOD) (μg/spot)	0.20	
Limit of quantification (LOQ) (μg/spot)	0.61	
**For Tablets**		
**Accuracy and Precision**		
Accuracy (*n* = 6)		
for 50% standard added (*n* = 6)	R = 99.0%; CV = 1.45%	
for 100% standard added (*n* = 6)	R = 99.2%; CV = 1.57%	
for 150% standard added (*n* = 6)	R = 99.8%; CV = 1.21%	
	Quantity of Precision (CV, (%)) *n* = 3	Acetylsalicylic Acid
Interday	8.00 μg/spot	0.78
	4.80 μg/spot	0.84
	1.60 μg/spot	1.73
Intraday	8.00 μg/spot	1.70
	4.80 μg/spot	1.56
	1.60 μg/spot	1.92
Robustness	Robust	

^a^ A = peak area (AU), x = amount (μg/spot) of drug analyzed, r = correlation coefficient, R = recovery (%), CV = coefficient of variation (%).

**Table 3:** The factors and their levels investigated in robustness test.

Symbol	Factors	Method Condition	Levels	
			+	−
X_1_	Chamber type	Twin Trough, 20 × 20 cm	Twin Trough, 20 × 20 cm	Twin Trough, 20 × 10 cm
X_2_	Sorbent type (E. Merck, #)	Al sheet (1.05570)	Al sheet (1.05570)	Al sheet (1.05554)
X_3_	Temperature of plate activation (°C)	120	130	110
X_4_	Extraction time (min)	30	31	29
X_5_	Volume of chloroform (mL)	5.0	5.1	4.9
X_6_	Volume of ethanol (mL)	4.0	4.1	3.9
X_7_	Saturation time of the chamber (°C)	20	22	18

**Table 4:** The experimental design matrix (2^3^) for the robustness test for the examined active substances in commercial effervescent tablets.

Experiment No			X_1_	X_2_	X_3_	X_4_	X_5_	X_6_	X_7_	Active Substance ^a^ Content (*y_i_*) (mg/tablet)	
										ASA	AA
1			+	+	+	+	+	+	+	503.5	207.2
2			+	+	−	+	−	−	−	494.5	182.2
3			+	−	+	−	−	+	−	506.5	200.4
4			+	−	−	−	+	−	+	482.5	199.6
5			−	+	+	−	+	-	-	519.0	207.0
6			−	+	-	-	-	+	+	510.5	204.0
7			−	−	+	+	-	-	+	473.5	192.2
8			−	−	-	+	+	+	-	494.0	198.2
Size of effect		ASA	2.500	17.750	5.250	13.250	3.500	11.250	11.000		
		AA	3.000	2.500	5.700	–7.800	8.300	7.200	3.800		
The label claim (mg)										500	200
Average amount (mg)										498.0	198.8
Variance										224.1	69.6
Standard devitation (SD)										15.0	8.3
Coefficient of variation (CV, %)										3.01	4.18

^a^ ASA = acetylsalicylic acid, AA = ascorbic acid.

**Table 5:** The statistical data concerning the results of the quantitative determination of active substances in combined effervescent tablets examined by NP-TLC with densitometry and the indirect iodometric methods.

	Active Substance ^a^ Determined by		
	NP-TLC with Densitometry		Indirect Iodometric Method
	ASA	AA	AA
Number of analysis	10	10	10
	487.8	204.6	203.2
	478.2	203.4	202.8
	469.8	201.6	202.0
	491.6	205.8	205.8
	476.3	201.8	201.6
	465.7	199.6	198.4
	502.2	205.6	200.6
	498.1	203.8	198.0
	489.2	201.8	198.2
	495.3	203.8	202.2
The label claim (mg)	500	200	200
Average amount (mg)	485.4	203.2	201.3
Minimum amount (mg)	465.7	199.6	198.0
Maximum amount (mg)	502.2	205.8	205.8
Variance	151.8	3.85	6.32
Standard deviation (SD)	12.3	1.96	2.51
Coefficient of variation (CV, %)	2.09%	0.96	1.25
Confidence interval of arithmetic mean with confidence level equal 95%	μ = 485.4 ± 8.8	μ = 203.2 ± 1.4	μ = 201.3 ± 1.8
Amount of active substance (%) in relation to the label claim	97.1%	101.6%	100.7%
TLC-Densitometric Method Compared with the Indirect Iodometric Method Determination of Ascorbic Acid			
	*t*-test	t calculated	1.89
		t_(95%.18)_ tabulated	2.101
	F-test	F calculated	1.64
		F_(95%.*f*1 = *f*2 = 9)_ tabulated	3.18

^a^ ASA = acetylsalicylic acid, AA = ascorbic acid.
